# Dynamic network participation of functional connectivity hubs assessed by resting-state fMRI

**DOI:** 10.3389/fnhum.2014.00195

**Published:** 2014-05-06

**Authors:** Alexander Schaefer, Daniel S. Margulies, Gabriele Lohmann, Krzysztof J. Gorgolewski, Jonathan Smallwood, Stefan J. Kiebel, Arno Villringer

**Affiliations:** ^1^Department of Neurology, Max Planck Institute for Human Cognitive and Brain SciencesLeipzig, Germany; ^2^Max Planck Research Group for Neuroanatomy & Connectivity, Max Planck Institute for Human Cognitive and Brain SciencesLeipzig, Germany; ^3^Department of High-field Magnetic Resonance, Max Planck Institute for Biological CyberneticsTübingen, Germany; ^4^Department of Psychology, The University of YorkHesslington, UK; ^5^Department of Neurology, Biomagnetic Center, University Clinics JenaJena, Germany; ^6^Berlin School of Mind and Brain, Mind and Brain InstituteBerlin, Germany; ^7^Department of Cognitive Neurology, University Hospital LeipzigLeipzig, Germany; ^8^Center for Stroke Research, Charité - UniversitätsmedizinBerlin, Germany

**Keywords:** graphs, self-generated thoughts, brain networks, mind wandering

## Abstract

Network studies of large-scale brain connectivity have demonstrated that highly connected areas, or “hubs,” are a key feature of human functional and structural brain organization. We use resting-state functional MRI data and connectivity clustering to identify multi-network hubs and show that while hubs can belong to multiple networks their degree of integration into these different networks varies dynamically over time. The extent of the network variation was related to the connectedness of the hub. In addition, we found that these network dynamics were inversely related to positive self-generated thoughts reported by individuals and were further decreased with older age. Moreover, the left caudate varied its degree of participation between a default mode subnetwork and a limbic network. This variation was predictive of individual differences in the reports of past-related thoughts. These results support an association between ongoing thought processes and network dynamics and offer a new approach to investigate the brain dynamics underlying mental experience.

## Introduction

One of the computational principles underlying behavior is that neuronal networks interact in a highly dynamic fashion (Dickinson, 1995; Marder and Bucher, 2001, 2007). While single neurons have been found to participate in multiple networks by means of the modulation of their synaptic connectivity (Hooper and Moulins, 1989; Weimann and Marder, 1994), it is an open question whether these dynamic events have an equivalent at the macroscopic, interregional level. Recent neuroimaging research in humans supports this hypothesis by demonstrating correspondence between large-scale brain networks and EEG microstates (Britz et al., 2010; Musso et al., 2010), which are transient, quasi-stable patterns in the EEG signal (Musso et al., 2010); as well as varying correlations between regions in the default-mode and task-positive networks (Chang and Glover, 2010). The existence of different large-scale brain states (Smith et al., 2012; Allen et al., 2014) suggests a spatially overlapping organization of specific areas. It is therefore conceivable that the dynamics of these regions allow brain areas to be members of multiple networks by varying their degree of membership over time.

One question facing cognitive neuroscience is how the full repertoire of cognitive capacities can be managed in a flexible manner. The finding of dynamic connectivity raises the possibility that temporal changes in brain connectivity may influence both mental states and behavior (Hutchison et al., 2013b; Allen et al., 2014). For example, the observation of a relationship between connectivity dynamics and heart rate variability provides support for an association to the current mental state (Chang et al., 2013). Other work has demonstrated that dynamic physiological measures such as pupillometry (Smallwood et al., 2012), the electroencephalogram (Barron et al., 2011), and changes in fMRI (Christoff et al., 2009; Smallwood et al., 2013) have all been linked to variations in mental state. A recent study found that alterations in current task performance are predicted by the extent of anti-correlation between the average signal of networks shortly preceding the task (Thompson et al., 2013). Furthermore, the flexibility of functional network configuration during a learning task has been shown to be predictive of later learning performance (Bassett et al., 2011). Given these findings, the hypothesis is that the dynamic interplay of different brain networks modulates ongoing thoughts or the current mental state. Ongoing thoughts during the resting-state can be assessed by a subsequent introspective self-report. Here, we want to examine if there is a relation between ongoing dynamics of functional connectivity and later self-reported thoughts.

Highly connected brain areas or hubs, which can be detected using structural (Hagmann et al., 2008; Gong et al., 2009) and functional (Buckner et al., 2009; Lohmann et al., 2010; Zuo et al., 2012) neuroimaging, have been shown to play a central role in whole brain communication (Sporns et al., 2007; Van den Heuvel and Sporns, 2013). Here, we hypothesized that multi-network hubs at the intersection of different networks may serve as dynamic relay stations to support communication between these networks as indicated by animal studies (Dickinson, 1995; Marder and Bucher, 2001, 2007). To examine this dynamic hypothesis, we tested if multi-network hubs keep their participation in each network at a constant level over time or rather dynamically change their degree of membership. We applied an edge clustering approach (Ahn et al., 2010) to cluster connectivity itself, thereby allowing regions to participate in multiple networks. The advantage of using this connectivity clustering algorithm is that we can directly assess the dynamically changing degree of participation of multi-network hubs in their networks. To address the relationship between changes in network dynamics and ongoing cognition, we tested whether the dynamics of hub participation varied across individuals with respect to the contents of thought that they reported at the end of the resting-state experiment.

In the context of resting-state connectivity dynamics, recent reports about the importance of BOLD signal variability (Garrett et al., 2011, 2013) are of relevance. As Garrett et al. could show the signal variability is not only reduced in poorer performance (Garrett et al., 2013), but is also further diminished in older subjects (Garrett et al., 2011, 2013). Here, we also tested for a link between aging and a reduction of network dynamics.

## Materials and methods

### Data and subjects

Data was acquired using a Siemens 3 Tesla Trio scanner and included resting-state functional magnetic resonance imaging (rs-fMRI) and a T1 anatomical scan. The rs-fMRI data were acquired over 900 volumes with 40 slices, a TR of 0.645 s and a resolution of 3 mm isotropic. The sequence (Xu et al., 2012) further comprised of the following parameters: *TE* = 30 ms, flip angle of 60° and a multiband factor of 4. Subjects were instructed to keep their eyes open and fixate on a crosshair. T1 anatomical scans were obtained using an MPRAGE sequence with a resolution of 1 mm isotropic.

From the initial 231 subjects we excluded 7 due to imaging artifacts and 44 for having maximum motion of more than 3 mm. To reduce potential micro motion artifacts we further removed 72 subjects with summed micro-movements (Van Dijk et al., 2012) above the group mean (0.1152 mm/volume). In addition, in the further analysis we still accounted for micro-movements as a covariate.

The resulting 108 subjects had a mean age of 37.71 years (std. 18.4 years) including 47 males and 61 females subjects. All data sets used in this study are part of the NKI Enhanced dataset (Nooner et al., 2012) and are made publicly available by the international neuroimaging data sharing initiative (Biswal et al., 2010). Institutional Review Board Approval was obtained at the Nathan Kline Institute and Montclair State University. Written informed consent was obtained for all study participants.

### Preprocessing

The preprocessing of resting-state fMRI data was carried out using FSL (Jenkinson et al., 2012), AFNI (Cox, 1996), and FreeSurfer (Dale et al., 1999). The steps included: (1) discarding the first four EPI volumes from each resting-state scan to allow for signal equilibration, (2) 3D motion correction, (3) slice time correction, (4) 4D mean-based intensity normalization, (5) removing linear trends, (6) regressing out 11 nuisance signals: six motion parameters and five top components from a principal components analysis of high variance signals (CompCor; Behzadi et al., 2007; Chai et al., 2012), and (7) band-pass temporal filtering (0.01–0.1 Hz). The output of these preprocessing steps is one 4D residual functional volume for each participant. In order to reduce partial volume effects no spatial smoothing was performed. We did not use global signal regression as the global signal is tightly coupled to the underlying neuronal signal (Schölvinck et al., 2010).

A non-linear transformation from T1 to a 3 mm isotropic MNI template (created from 152 subjects, provided with FSL) was calculated for individual T1 images using ANTs (Avants et al., 2011). This transformation was combined with the EPI to T1 transformation (bbregister Greve and Fischl, 2009) to warp the EPI volumes to standard MNI space.

The preprocessing pipelines used for this manuscript can be downloaded from https://github.com/alexschaefer83/DynamicHubs and used together with BIPS (https://github.com/INCF/BrainImagingPipelines) which is based on nipype (Gorgolewski et al., 2011).

### Graph construction

A graph is an abstract representation of a network. A graph G consists of a set of vertices V and a set of edges E. An edge indicates the presence of a relationship between two vertices, which in case of this study is functional connectivity. We will also refer to edges as *connections*. To account for different strength of functional connectivity we use an edge weighted graph.

For the graph construction we parcellated the functional images into 200 cortical and subcortical regions. The parcellation is based on spatially constrained spectral clustering (Craddock et al., 2012) which aims to create spatially coherent regions of functional homogenous connectivity. The parcellation is publicly available (www.nitrc.org/projects/cluster_roi). In the proposed analysis we used the version derived from the best performing clustering in the publication by Craddock et al. (2012) (two-level clustering with the rt similarity metric). Each parcel is one unique vertex in our graph. To estimate the relationships between the vertices, the average signal within each parcel was extracted and its pairwise correlation with the signals (spatial averages over all voxels of a parcel) of all other parcels computed. The resulting correlation values were Fisher z-transformed in order to allow for an unbiased analysis in the further steps. We then averaged the *z*-values over all subjects. For the graph we assigned an edge (or connection) between two vertices (or parcels) if their respective correlation value belonged to the highest 10% (2000 edges) in the group average. Furthermore, we weighted the edge by the corresponding *z*-value. This technique shows good reliability (Schwarz and McGonigle, 2011) as it incorporates only strong connections with relatively high reliability (Patriat et al., 2013). Visualization of brain graphs was performed using braingl (Böttger et al., 2014) and conview (http://conview.googlecode.com).

### Connectivity clustering

**Figure 1:**
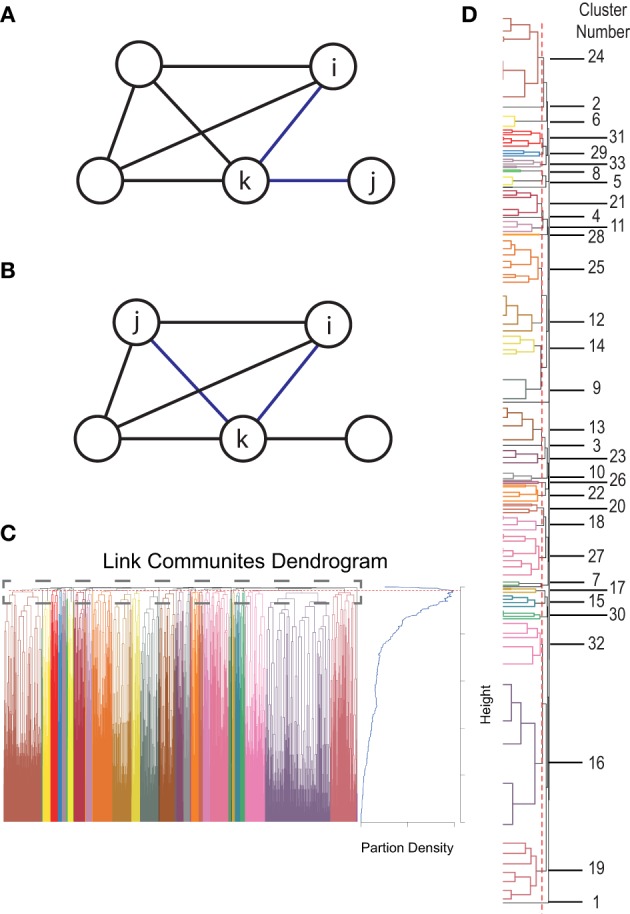
**Connectivity clustering approach. (A)** Example for two connections with low similarity. The common neighborhood of vertices *i* and *j* is only *k* therefore the similarity is *S* = 1/3. **(B)** An example for two connections with high similarity. As vertices *i* and *j* have the same neighborhood the similarity is 1. **(C)** Dendrogram (left) based on calculated similarities together with a plot of estimated partition densities (right). Red dotted line indicates the cutoff of the dendrogram estimated by maximizing partition density. **(D)** Zoomed view of the top of the dendrogram from **(C)** together with network numbers from Figure 3.

An efficient way to cluster connections has been proposed by Ahn et al. (2010). We used an implementation of this idea by Kalinka and Tomancak (2011). In order to cluster connections one requires a measurement of their similarity. Ahn et al. (2010) proposed the Jaccard coefficient to estimate the similarity between connections *e*_*ik*_ and *e*_*jk*_ that share a vertex *k*:

$$S\left(e_{ik},e_{jk}\right)=\frac{\left|n(i)\cap n(j)\right|}{\left|n(i)\cup n(j)\right|}$$

where *n*(*i*) and *n*(*j*) is the first order neighborhood of vertex *i* and *j*, respectively. An example for two connections with low similarity is illustrated in Figure 1A. The similarity is *S* = 1/3 as the common neighborhood of vertices *i* and *j* is only vertex *k*. An example for two connections with high similarity (*S* = 1) is illustrated in Figure 1B as *i* and *j* have the same neighborhood. To better account for the different strength of connections we use a weighted version called Tanimoto coefficient:

$$S\left(e_{ik},e_{jk}\right)=\frac{w_{i}w_{j}}{{\left|w_{i}\right|}^{2}+{\left|w_{j}\right|}^{2}-w_{i}w_{j}}$$

where **w**_*i*_ is a vector describing the weights of the connections between vertex *i* and the vertices in the first order neighborhood of *i* and *j*. After calculating the pairwise Tanimoto coefficients between all links in the network, a hierarchical clustering is performed using McQuitty's similarity method (McQuitty, 1966). The optimal cutoff for the resulting dendrogram (tree diagram, Figure 1C) is determined by maximizing the partition density (Ahn et al., 2010). This is the density within the clusters, normalized for the maximum and minimum number of possible connections within each network. More explicitly, for a network with *M* connections, {*P*_1_, …, *P*_*C*_} is a partition of the connections into *C* subsets. A subset *P*_*c*_ has *m*_*c*_ = |*P*_*c*_| connections and $n_{c}=\left|\cup_{e_{ij\in P_{c}}}\left(i,j\right)\right|$ vertices. Then Ahn et al. define the partition density of a subset *C*:

$$D_{c}=\frac{m_{c}-\left(n_{c}-1\right)}{\frac{n_{c}\left(n_{c}-1\right)}{2}-\left(n_{c}-1\right)}$$

This is *m*_*c*_ normalized by the minimum and maximum numbers of connections possible between *n*_*c*_ vertices. The partion density *D*, is the average of *D*_*c*_, weighted by the fraction of present connections:

$$D=\frac{2}{M}\sum_{c}m_{c}\frac{m_{c}-\left(n_{c}-1\right)}{\left(n_{c}-2\right)\left(n_{c}-1\right)}$$

The maximum partition density gives the optimal cutoff for the dendrogram which determines the number of connectivity clusters in our solution (Figure 1D).

### Reliability of clustering

To evaluate the reliability of the new clustering method we use a split half test. In order to not compare clustering over different elements (connections) we used the same connections as in the full group for both clusterings (see Graph Construction). To evaluate reliability of the cluster results we divided our subjects into two groups, one of subjects with odd index and one of subjects with even index, and performed the clustering for both of them separately. As distance between the two found clustering solutions (even and odd index) we computed the so-called cophenetic correlation coefficient (Sokal and Rohlf, 1962). To compute confidence intervals for the results we performed a mantel statistic using 999 permutations (Mantel, 1967) which creates a permutation baseline. The two found clustering solutions correlated with *r* = 0.8954 (*p* < 0.001, 95% CI = 0.001185). The similarity of the results across subgroups implies a certain generalizability of our cluster results across a larger population.

### Windowing of temporal dynamics

To investigate the underlying dynamics of the resulting connectivity clusters instead of correlating signals over the whole scan session, a shifting time window is used:

$$r_{t}=corr\left(x\left(t\cdot w..\left(t+1\right)\cdot w-1\right),y\left(t\cdot w..\left(t+1\right)\cdot w-1\right)\right)$$

where *t* is the timepoint with *t* = 1..11, *w* is the window with *w* = 77 (or 49, 7 s) and *x*, *y* are the time series of two (out of 200) parcels. All estimated *r*-values are Fisher's *z*-transformed, resulting in 11 *z*-values per connection and subject. Each of these 11 values is adjusted to a within subject baseline by subtracting from the estimated *z*-value for the complete scan time. These normalized values reflect, for each subject, the temporal change of the connection strength over the 11 time intervals.

### Analysis of temporal dynamics

**Figure 2:**
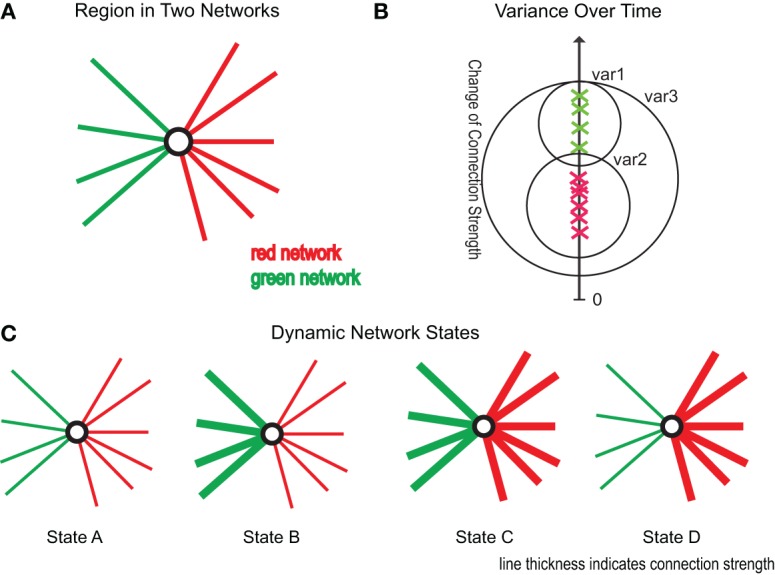
**Illustration of research ideas. (A)** One region as part of two connectivity clusters (red and green). **(B)** Green crosses mark connectivity change of connections within the green cluster, the red crosses of connections within the red cluster. The y-axis gives the amount of change of the respective connections. var1, var2 are the variances within the red and green cluster, while var3 gives the variance over all connections. **(C)** Four possible scenarios of changing connectivity: State A: reduction of both clusters, State B: reduction of red cluster and increase of green cluster, State C: increase of both clusters, and State D: reduction of green cluster and increase of red cluster.

To investigate if the connections within a connectivity cluster change, over time, more similarly than over connectivity clusters we measured the variance of connectivity change within (var1, var2 in Figure 2B) and without clusters (var3 in Figure 2B). Timepoints were not averaged over subjects as subjects are likely in a different state of dynamic functional connectivity and therefore not comparable. We tested whether the within cluster variance was significantly smaller to the variance over clusters using a Wilcoxon test.

To analyze if regions in multiple networks Figure 2A show a dynamically varying degree of membership between its belonging networks we used the following strategy. To test if a region changes the degree of membership between its networks, we estimated the average strength of connections assigned to one network and compared it to the average strength of connections belonging to another network using a dynamic windowing (50 s) approach. These “variation” events are illustrated as State B and State D scenario in Figure 2C and can be well-captured by the mean squared error (MSE) between the average correlation strengths. To test the significance of our results we used permutation testing based on random clustering. In this clustering we chose randomly connections from the same hub regions without caring about assigned cluster values. The number of connections was equal to the original clusters. We created 10,000 times two corresponding random clusters and computed the MSE between them. The results are plotted in Figure 5C.

For the whole brain analysis we aimed at ensuring that networks tested included enough connections for a stable signal. Therefore, we chose only regions where the second largest network included at least two connections (151 of 200 regions, Figure 4C). The MSE was then averaged over all of pairs of networks which shared participation of the particular hub.

### Experiment: self-generated thoughts

To assess thoughts and feelings during the scan the subjects were interviewed directly after MRI session using the New York Cognition Questionnaire (NYC-Q). The beginning of the interview was approximately 45 min after the resting state scan that we analyzed. The NYC-Q is a self-report tool consisting of two sections, the first containing questions about the content of thought (23 questions), the second containing questions about the form that these thoughts take (8 questions). In each question subjects were asked to indicate how well a statement described their thoughts on a scale from 1 (“Completely did not describe my thoughts”) to 9 (“Completely did describe my thoughts”). Therefore, a high score on a component relates to the subjective experience of mind-wandering which also implies that one strong thought yield a similar score as multiple seemingly insignificant thoughts. We used the data and code available online (https://github.com/NeuroanatomyAndConnectivity/NYC-Q) provided and described in greater detail by Gorgolewski et al. (in press). The 23 answers about the content of thought collected from 166 subjects were factorized into five categories. The factorization was performed using principal axis factor analysis together with an oblimin rotation (Revelle, 2011) to increase interpretability. The number of factors was estimated using Parallel Analysis (Horn, 1965). Individual-level scores were computed applying the method by Ten Berge et al. (1999). We used the names and interpretation of these categories as described in the original study, namely thoughts about the past (Past), the future (Future), positive thoughts (Positive), negative thoughts (Negative), or thoughts about relationships (Social Cognition). The eight questions about the form of thought were factorized into three factors as described above. The factors were named as in the original study: in form of words (Words), in form of images (Images), and specificity of words (Vague). As the factorization employed does not enforce orthogonality of the components, we performed for each component a partial correlation analysis with the respective other seven components as covariates. A more detailed description about NYC-Q and the factorization employed can be found in the study by Gorgolewski et al. (in press).

## Results

### Hub regions belong to multiple networks

**Figure 3:**
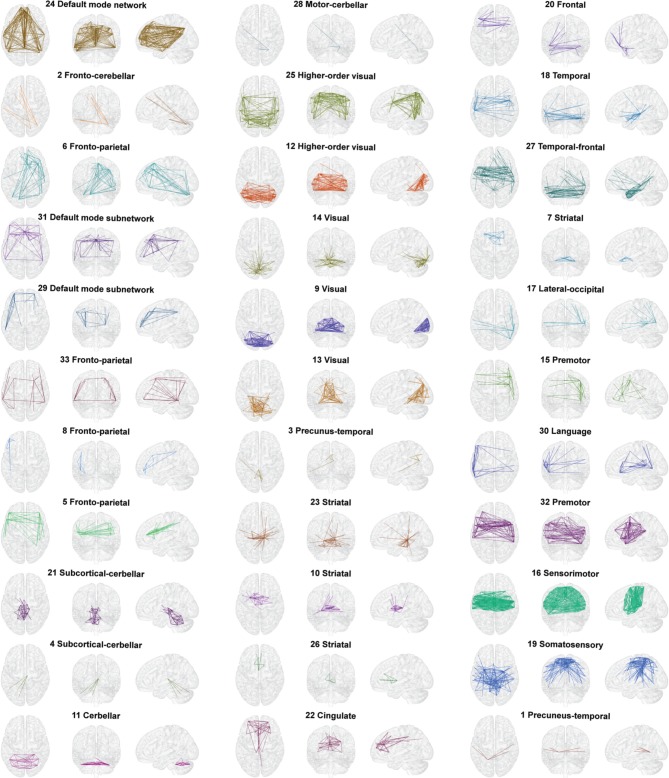
**Connectivity networks**. All 33 connectivity networks found in the hierarchical cluster analysis of time and group averaged connectivity. Networks can also be inspected interactively and in three dimensions online (http://openscience.cbs.mpg.de/schaefer).

**Figure 4:**
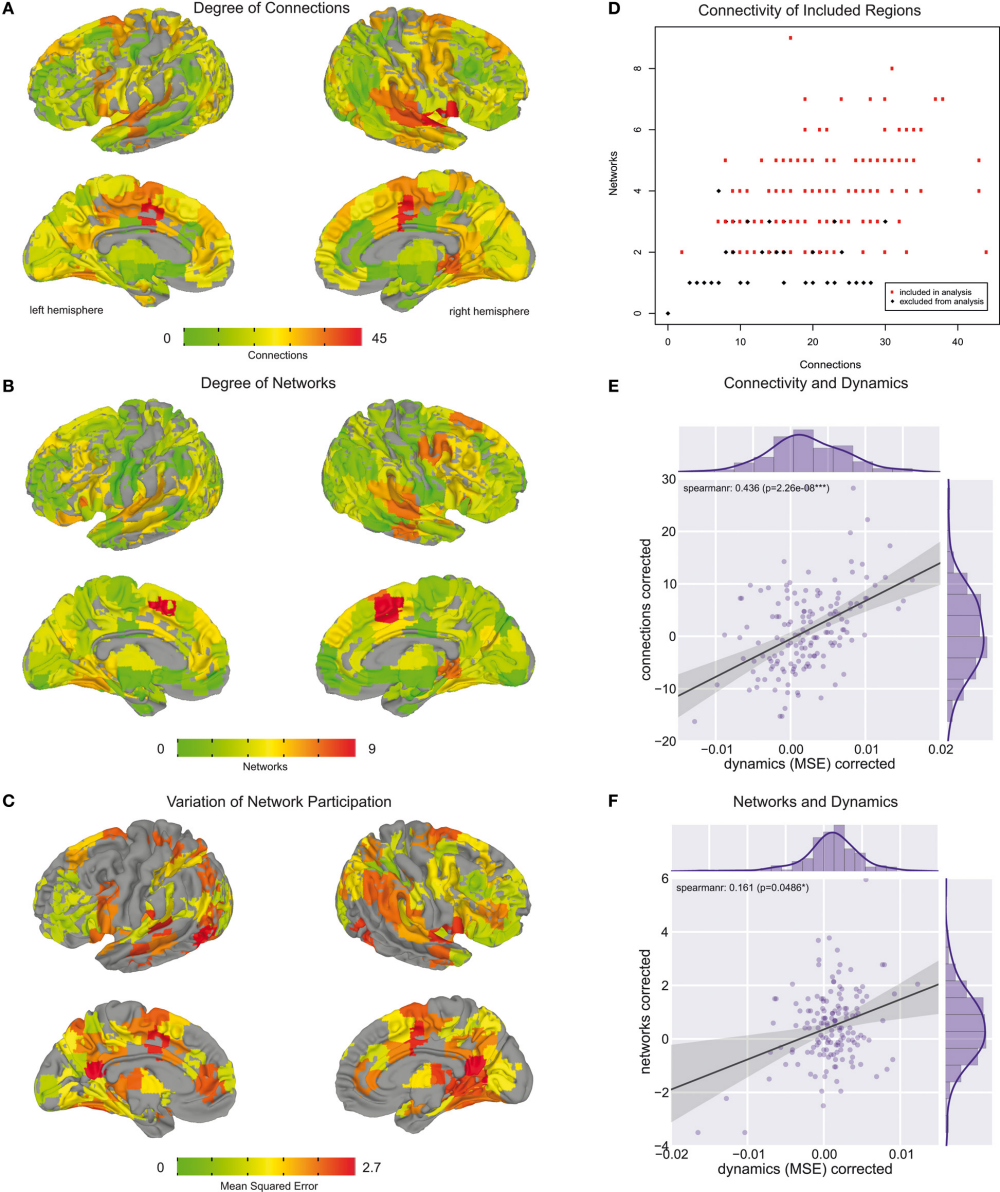
**Whole brain regional overview. (A)** Brain areas colored by their degree of connectivity. Areas with more connections are displayed in red, whereas regions with fewer connections are in green. **(B)** Brain areas colored by the number of networks they are part of. Regions participating in a higher number of networks are displayed in red. Regions which are part of fewer networks are depicted in green. **(C)** Brain areas colored by their variation of network participation. Areas with higher variation are colored in red, whereas regions with lower variation are shown in yellow and green. **(D)** Relation between number of connections and number of networks each brain region is part of. In the further analysis we only included regions which are part of at least two networks and for which the second largest belonging network consisted of at least two connections. These regions are colored in red. **(E)** Relation between number of connections and variation of network participation measured across brain areas included in analysis. **(F)** Relation between number of networks and variation of network participation measured across brain areas included in analysis.

Using the edge clustering approach described above (Ahn et al., 2010) we found 33 networks (Figure 3), several of which are well-characterized networks typically observed in fMRI resting-state experiments. The clusters can be downloaded from https://github.com/alexschaefer83/DynamicHubs or interactively viewed online http://openscience.cbs.mpg.de/schaefer. The number of connections and regions in each network can be found in Table 1. We found that the majority of regions (174 of 200) participated in more than one network. An overview of the amount of multi-network participation is given in Figure 4B. In contrast Figure 4A gives the degree of connectivity of these regions. The relation of the two measures connectivity and multi-network participation is given in Figure 4D.

**Table 1:** **Descriptive information of connectivity networks (Figure 3)**.

**Network**	**Vertices (regions)**	**Edges (connections)**
1	5	8
2	6	6
3	6	8
4	5	4
5	16	33
6	18	46
7	8	18
8	6	8
9	19	105
10	16	36
11	14	35
12	24	109
13	31	92
14	34	49
15	21	30
16	33	369
17	16	16
18	32	41
19	59	147
20	19	20
21	20	64
22	21	42
23	28	41
24	26	217
25	38	111
26	7	7
27	34	106
28	4	3
29	14	23
30	19	26
31	25	41
32	31	114
33	20	25
Average	20.5	60.6

*Connectivity networks together with their respective number of vertices (regions) and number of edges (connections)*.

### Hub regions vary degree of membership between networks

**Figure 5:**
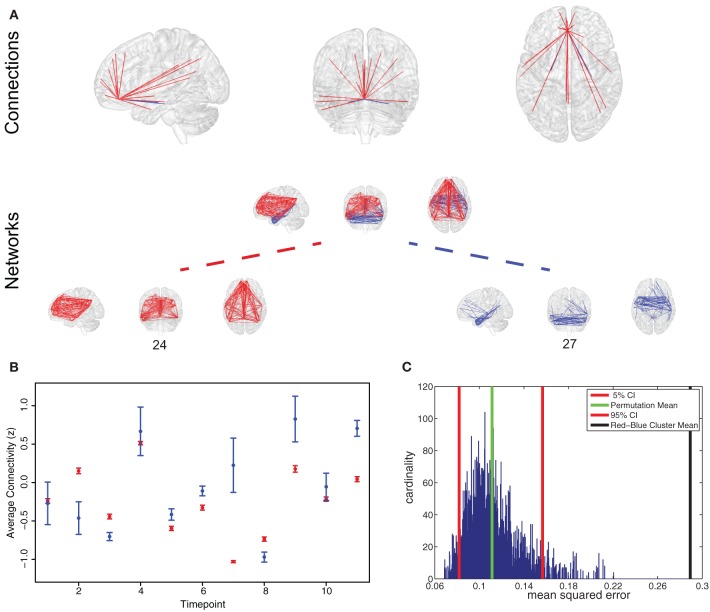
**Representative region for variation of network participation. (A)** Anterior cingulate cortex (ACC) participating in two networks (networks 24 and 27). **(B)** Example plot of ACC participation in the two networks in a 50 s interval. Timepoint 2 and 7 are good examples of a variation in network participation (State B or State D, Figure 1C). **(C)** Permutation test shows that variation behavior measured by mean squared error is more pronounced in the found clustering than in a random clustering.

As an example Figure 5 presents the results for the analysis on the anterior cingulate cortex (ACC). While an overview for whole brain results is given in Figure 4C, we chose the ACC as a representative area to illustrate the typical results for a single area. We found the ACC to participate in two spatially separated networks: a temporal network and a frontal-parietal network (Figure 5A).

To test if connectivity changes occur more within clusters than between clusters we estimated the variance of connectivity change across timepoints and subjects. We found the variance of change within the red cluster (*p* = 0.0181) and within the blue cluster (*p* = 1.156 * 10^−23^) to be smaller than the variance of connectivity change over both clusters.

To test if a region changes the degree of membership between its networks, we estimated the average strength of ACC connections assigned to one network and compared it to the average strength of ACC connections belonging to another network. As shown in Figure 5B, we found changes in the degree of membership, which we quantified by the mean squared difference in participation. We used permutation tests to show that these changes between networks were statistically significant, see Figure 5C. We found there were significantly stronger changes in the degree of participation between specific clusters than would be expected between randomly selected clusters (Figure 5C).

A whole-brain overview of the amount of variation in network participation can be seen in Figure 4C. We also investigated if there is a relation between dynamic (Figure 4C) and connectivity-hubness (Figure 4A) or network-hubness (Figure 4B). We found a positive correlation (Spearman *r* = 0.44, *p* = 2 * 10^∧^ − 8, *n* = 151) between variation of network participation and the degree of connectivity (Figure 4E) while we accounted for the number of networks. Further, we also found a weaker correlation (Spearman *r* = 0.16, *p* = 0.049, *n* = 151) between the dynamics of a region and the amount of networks the region participates in (Figure 4F) while accounting for the number of connections.

### Association between variation of network participation and self-generated thoughts

**Figure 6:**
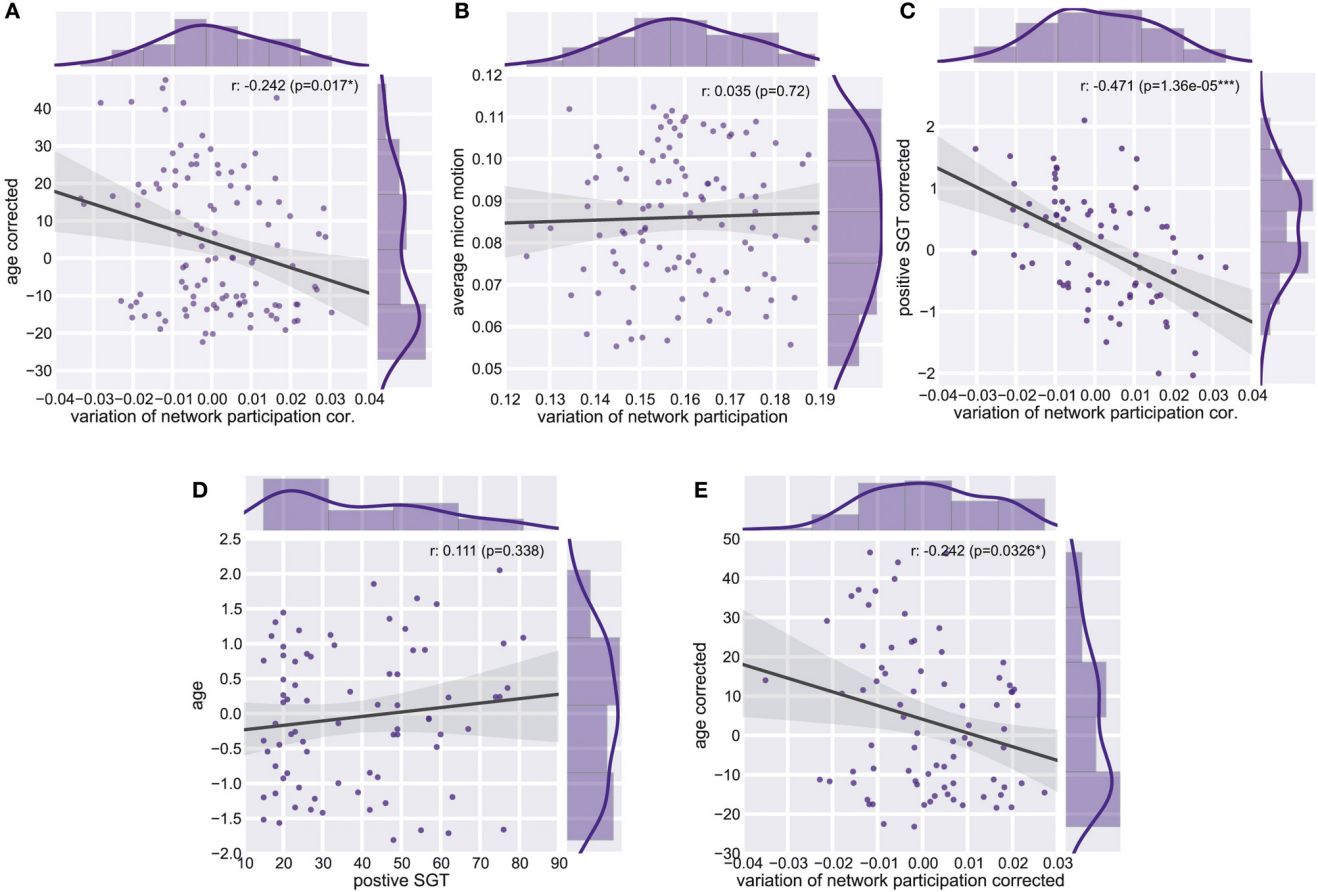
**Whole Brain Variation of Participation. (A)** Partial correlation between average variation of participation and age corrected for micro-movements. **(B)** Correlation between average variation and average micro-motion. **(C)** Partial correlation between average variation of participation and positive self-generated thoughts (SGT) corrected for micro-movements, age, gender, and the respective other four factors of SGT. **(D)** Correlation between age and positive self-generated thoughts (SGT) corrected for micro-movements. **(E)** Partial correlation between average variation of participation and age corrected for positive self-generated thoughts (SGT), micro-movements.

**Figure 7:**
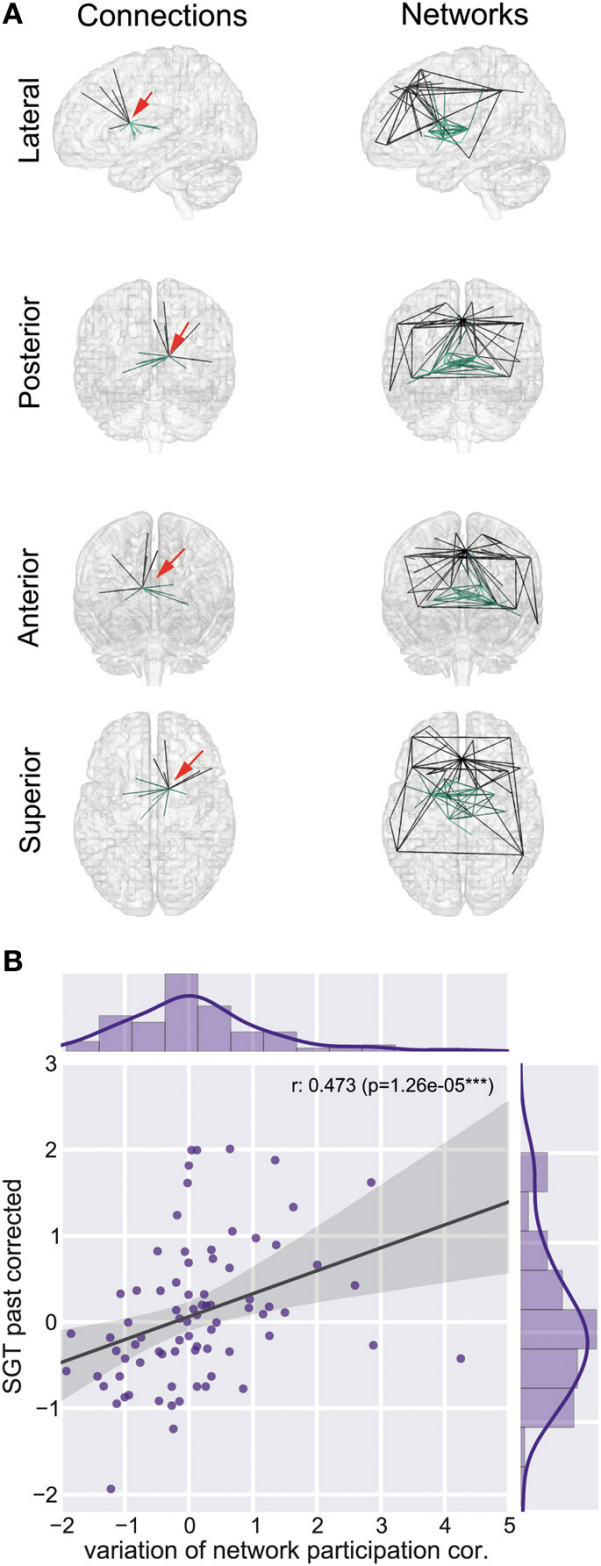
**Left caudate region and self-generated thoughts about past events. (A)** Left caudate region is part of two connectivity networks, a sub cortical network (network 10) and a subnetwork of the default mode network (network 31). Red arrow indicates the position of the left caudate region. **(B)** Increased correlation between variation of network participation of the left caudate region and self-generated thoughts about past events.

We found a reduction of whole brain-averaged variation of network participation in subjects which reported to have more positive thoughts during the scan [Figure 6C, Spearman *r* = −0.47, *p* = 0.0001 (corrected for 8 comparisons), *n* = 78]. An overview of whole brain participation variation is also given in Figure 4D. We further investigated if the variation of network participation of a specific region correlates with a domain of self-generated thoughts. We found an increased variation of network participation of the left caudate in subjects which reported to have more thoughts about the past during the scan {Figure 7, Spearman *r* = 0.47, *p* = 0.02 [corrected for 1600 comparisons (8 categories * 200 regions)], *n* = 78}.

### Decrease of dynamics in age

As the variability of the fMRI signal has been found to decrease in aging (Garrett et al., 2011, 2013), we tested whether similar effects can be found for the dynamic of network participation of brain regions between networks. Critically, we found a significant age effect on connectivity changes across the whole brain (spearman *r* = −0.24, *p* = 0.011, *n* = 106, Figure 6A). While we used micro-movements as a covariate we also tested for a relationship between network dynamics and micro-movements. However, we did not find a relation between whole brain dynamics and micro-movements (*r* = 0.03, *p* = 0.747, *n* = 106, Figure 6B). We found no significant correlation between age and positive self-generated thoughts (SGT) corrected for micro-movements (*r* = 0.111, *p* = 0.338, *n* = 78, Figure 6D). However, when we added positive SGT as a covariate the negative correlation between age and participation remained similar (spearman *r* = −0.24, *p* = 0.033, *n* = 78, Figure 6E).

The impact of different initial graph thresholds onto the three main results of this paper are shown in Table 2. In this manuscript we used a threshold of 10% or 2000 connections. All results remained significant for thresholds from 7.5% (1500 connections) to 15% (3000 connections). For a threshold of 17.5% (3500 connections) the caudate finding became insignificant. The clustering of connectivity for sparsity thresholds of 20% and higher resulted in a single network, whereas for sparsity threshold of 5% and lower the left caudate was part of a single network. In this context no effect sizes were calculated.

**Table 2:** **Impact of different initial graph thresholds for the main results of the manuscript**.

**Threshold**	**Whole brain VNP/positive SGT (*r*)**	**whole brain VNP/age (*r*)**	**Caudate VNP/past SGT (*r*)**
17.5% (3500 edges)	−0.46	−0.21	0.14
15% (3000 edges)	−0.43	−0.26	0.41
12.5% (2500 edges)	−0.49	−0.20	0.38
10% (2000 edges)	−0.47	−0.24	0.47
7.5% (1500 edges)	−0.46	−0.21	0.40

*The threshold of 10% (2000 edges) was used in this study. Main results are partial correlation between whole brain averaged Variation of Network Participation (VNP) and positive Self-Generated Thoughts (SGT), whole brain averaged VNP and age, VNP of left caudate and SGT about the past*.

## Discussion

We have shown that multi-network hubs vary their degree of participation into different networks over time. In addition, we found that these network dynamics were inversely related to age and to positive self-generated thoughts reported by subjects. These results demonstrate a novel analytic approach which enables a testable framework for quantifying dynamic network interaction across regions on an individual-level.

To facilitate this analysis we used an algorithm to cluster connectivity itself rather than brain regions based on their connectivity profiles (Power et al., 2011). This approach offers the advantage to maintain whole brain information in connectivity space rather than reducing it to the regional space. This is important as we are interested in functional brain networks which includes connections and regions rather than only regions (Damoiseaux et al., 2006; Power et al., 2011). This domain shift also includes the conceptual advantage that brain regions can participate in multiple networks. While the later advantage is shared by other network decomposition methods which allow networks to overlap like independent component analysis (ICA, Calhoun et al., 2001; Beckmann et al., 2005), non-negative matrix factorization (NMF, Lee et al., 2011), and Latent Dirichlet Allocation (LDA, Yeo et al., 2014) it is still conceptually different as these networks exist in the spatial location but not in the connectivity space.

One of the limitations of connectivity clustering is that an initial arbitrary threshold onto the connectivity has to be applied. While we found that the effect sizes of our main results are comparable at a range of thresholds 7.5 to 15% (Table 2) we also found that for a threshold of the 17.5% the relation between caudate switching and past self-generated thoughts was insignificant. One reason for this might be the higher connectedness of the left caudate at this threshold which makes the left caudate a part of 10 networks, in contrast to two networks at 10% sparsity. For even higher thresholds the network clustering resulted in a single network. In this context a threshold between 7.5 and 15% seems recommendable for similar future analyses.

While brain regions in our framework are allowed to participate in multiple networks we tested the hypothesis that this participation is not necessarily static over time. To illustrate this using the example of the ACC, Figure 5 shows a region in the ACC which takes part in two networks (networks 24 and 27). The [blue] network is spatially similar to the medial temporal lobe subsystem of the default mode networks (DMN), as described by Andrews-Hanna et al. (2010) and the [red] network is spatially similar to the dorsal medial prefrontal cortex subnetwork of the DMN (Andrews-Hanna et al., 2010). However, we found that the participation of the ACC region in these two subnetworks is not static over time (Figure 5B). This offers further interpretation of how these two subnetworks may interact in the DMN: the affiliation of this portion of ACC varies between them over time. Additionally, the variation network participation in single brain regions may in part account for the diversity of functions often associated with hub regions (Cole et al., 2013) such as the ACC (Devinsky et al., 1995).

While a recent primate study has shown dynamic of functional connectivity even in the absence of consciousness (Hutchison et al., 2013b), more recent human studies show relations of dynamic functional connectivity and physiology (Chang et al., 2013) as well as task performance (Thompson et al., 2013). Here we expand this picture by showing a relationship between dynamic functional connectivity and ongoing thought processes.

We found that variation in network participation was correlated with self-generated thought content across individuals; specifically it was related to thoughts about the past and those with a positive tone. Neurobiological studies suggest that there exist at least two different memory systems, one more cognitive system which relies upon medial temporal lobe and hippocampus areas and a stimulus-response system which banks in the basal ganglia (Poldrack and Packard, 2003). The default-mode network is a cortical network that connects middle temporal with the posterior cingulate and prefrontal areas, the subcortical network associated with the left caudate connects subcortical regions such as thalamus, putamen, and caudate. Here we found that the left caudate is a hub involved in varying its degree of participation between these two networks and its specific behavior was correlated with self-generated thoughts about past events.

Self-generated thoughts about the past are known correlates of unhappiness (Smallwood and O'Connor, 2011) and may mark the temporal precursor of negative mood (Ruby et al., 2013). Self-generated thoughts of the past are also accompanied by greater disengagement, or decoupling, from external processes, as indexed by worse task performance (Smallwood et al., 2007, 2009). Altogether these studies illustrate that retrospective self-generated experiences may at times be both intrusive and unpleasant. The heightened variation in network participation of the caudate nucleus with increasing retrospective focus could therefore reflect the greater competition that accompanies especially repetitive or intrusive self-generated thoughts. Broadly consistent with this account we found that positive thoughts were associated with less variation in network participation at a whole brain level. In contrast to retrospective thoughts, pleasant experiences were associated with more consistent network dynamics, possibly reflecting the relatively calm form that positive experiences may take.

Variability of the fMRI signal, an univariate measure of brain dynamics, has been found to play an important role in behavioral performance (McIntosh et al., 2010; Garrett et al., 2013). One crucial observation is its reduction in older individuals or those who performing poorly in a variation of cognitive tasks (Garrett et al., 2013). While the biological reason for the loss of variability might arise from a dysregulation of dopamine and glutamate (Hong and Rebec, 2012), the behavioral implications might be explained by computational models (Deco et al., 2011). These models suggest that variability of brain signal is important for exploring the repertoire of possible brain states (Deco et al., 2011), while lower variability will raise the potential for remaining in a single state—resulting in inflexible behavior. Here we have shown a decline of variation in network participation during aging. Aging is therefore not only associated with decreased signal variability but also reduced interplay between networks, suggesting that dynamic network participation may underlie behavioral flexibility. However, the interaction of dynamics and age was not linear, indicating a more complex relation between dynamics and age which might be investigated in future studies.

Interactions of networks have been studied to date with respect to their average signal. The anticorrelation between the average signal of the DMN and the average signal of the task positive network has been found to be predictive of individual behavior during task and rest (Kelly et al., 2008). Recently it has been shown that this relationship changes during the task based on the current performance (Thompson et al., 2013). A relationship between this anticorrelation and variation in network participation is possible, but not straightforward. As anticorrelation is based on the average signals of all vertices in respective networks the variation in network participation describes the varying integration of single regions into the networks.

In the analysis of dynamic network participation we only included regions which belonged to two or more networks and where the second largest consisted of at least two connections (Figure 4D). While this property is fulfilled by all highly connected regions (Figure 4D), there were also few sparsely connected areas included into the analysis. In this context we focused stronger on multi-network hubs then their pure connectedness, while we also found a correspondence between these two properties (Figure 4D).

While we hypothesized that dynamic network interaction takes place in highly connected areas we also found that the extent of these dynamics is related to the extent of the hubness (Figure 4E). This evidence further supports our hypothesis that hub areas might serve as relay stations which enable information integration. However, the effect of multi-network hubness was much weaker (Figure 4F) and more connections could just improve the detectability of the underlying dynamic process.

The description of dynamic organization in resting-state connectivity (Majeed et al., 2009; Britz et al., 2010; Chang and Glover, 2010; Musso et al., 2010; Handwerker et al., 2012; Smith et al., 2012) raised concerns about the potential artifactual origin of these BOLD synchrony fluctuations. Studies using EEG-fMRI data, however, have established a neuronal origin of dynamic resting-state connectivity (Britz et al., 2010; Musso et al., 2010; Tagliazucchi et al., 2012). Another recent functional connectivity study in anesthetized macaques demonstrated dynamic functional connectivity in the absence of any motion (Hutchison et al., 2013b). In order to avoid potential confounds in our study, we selected datasets with minimal motion and tested for remaining influences. We did not find that subject motion accounted for the increased variation in network participation (Figure 6B).

A limitation of the current study is the fixed window length of 50 s which we chose based on the size of our bandpass filter (10 s to 100 s). This length might not always coincide with the dynamic of ongoing cognitive processes. To give evidence for the robustness of our findings we reanalyzed the data with window lengths of 65 s (100 volumes). All our main results remained significant. However, a data-driven approach to detect temporal change points as described recently by Cribben et al. (2012) gives an adaptive window length which could further enhance sensitivity to dynamic processes. A potentially confounding influence is the variation of node sizes which could affect the extracted time series differently by noise. While the parcellation used in this study aimed to reduce the variation of node sizes (Craddock et al., 2012, Table 2) a potential influence cannot be ruled out. A limitation in the current study design is the correlational approach to relate behavior and resting-state dynamics. We cannot rule out that an unknown third variable may have caused the observed effects. To what extent a manipulation of the content of self-generated thoughts could affect the dynamics of functional connectivity requires further research.

Understanding the dynamics of brain (Smith et al., 2012; Hutchison et al., 2013a; Allen et al., 2014) and mental states (Smallwood, 2013) is thought to be important as it may explain the flexible way that cognition unfolds over time. We identified dynamics in network participation for brain regions that occurred at multi-second time-scales which are correlated with alterations in self-reported experience. Although traditional functional connectivity approaches have until recently often ignored fluctuations over time, our findings suggest that understanding the dynamics of cognition may depend upon methods that interrogate the complexity and flexibility of brain dynamics. Our demonstration of a relation between the dynamic variation in network participation of brain regions and psychological measures of experience, therefore, indicates that understanding the temporal brain dynamics could illuminate the processes through which different characteristics of self-generated experience arise.

### Conflict of interest statement

The authors declare that the research was conducted in the absence of any commercial or financial relationships that could be construed as a potential conflict of interest.
